# Dosimetric comparison of extended dose range film with ionization measurements in water and lung equivalent heterogeneous media exposed to megavoltage photons

**DOI:** 10.1120/jacmp.v4i1.2539

**Published:** 2003-01-01

**Authors:** Paule M. Charland, Indrin J. Chetty, Shigeru Yokoyama, Benedick A. Fraass

**Affiliations:** ^1^ Department of Radiation Oncology The University of Michigan Ann Arbor Michigan 48109‐0010

**Keywords:** film dosimetry, lung, photon beam dosimetry

## Abstract

In this study, a dosimetric evaluation of the new Kodak extended dose range (EDR) film versus ionization measurements has been conducted in homogeneous solid water and water‐lung equivalent layered heterogeneous phantoms for a relevant range of field sizes (up to a field size of $25\times 25{\;\text{cm}}^{2}$ and a depth of 15 cm) for 6 and 15 MV photon beams from a linear accelerator. The optical density of EDR film was found to be linear up to about 350 cGy and over‐responded for larger fields and depths (5% for $25\times 25{\;\text{cm}}^{2}$ at depth of 15 cm compared to a $10\times 10{\;\text{cm}}^{2}$, 5 cm depth reference value). Central axis depth dose measurements in solid water with the film in a perpendicular orientation were within 2% of the Wellhöfer IC‐10 measurements for the smaller field sizes. A maximum discrepancy of 8.4% and 3.9% was found for the $25\times 25{\;\text{cm}}^{2}$ field at 15 cm depth for 6 and 15 MV photons, respectively (with curve normalization at a depth of 5 cm). Compared to IC‐10 measurements, film measured central axis depth dose inside the lung slab showed a slight over‐response (at most 2%). At a depth of 15 cm in the lung phantom the over‐response was found to be 7.4% and 3.7% for the $25\times 25{\;\text{cm}}^{2}$ field for 6 and 15 MV photons, respectively. When results were presented as correction factors, the discrepancy between the IC‐10 and the EDR was greatest for the lowest energy and the largest field size. The effect of the finite size of the ion chamber was most evident at smaller field sizes where profile differences versus film were observed in the penumbral region. These differences were reduced at larger field sizes and in situations where lateral electron transport resulted in a lateral spread of the beam, such as inside lung material. Film profiles across a lung tumor geometry phantom agreed with the IC‐10 chamber within the experimental uncertainties. From this investigation EDR film appears to be a useful medium for relative dosimetry in higher dose ranges in both water and lung equivalent material for moderate field sizes and depths. © *2003 American College of Medical Physics*.

PACS number(s): 87.53.Dq, 87.66.Cd, 87.66.Jj, 87.66.Xa

## INTRODUCTION

The acquisition of accurate measured dosimetry data in inhomogeneous conditions is fundamental not only in evaluating the perturbative effect of inhomogeneities but also in verifying or validating robust calculational algorithms. Suggested dose accuracy for a treatment planning system (1–4% for a simple homogeneous phantom^1^) often needs to be arbitrarily relaxed in inhomogeneous situations depending on the sophistication of the algorithm. Accurate dose calculation becomes particularly important, however, in the context of dose escalation in the lung^2^ and study of lung complication probabilities.^3^

Many investigators have shown the existence of significant dose perturbation within, and beyond, low‐density inhomogeneities for small fields of megavoltage photons.^4^–^7^ The perturbations in lung result from the combined effects of a reduction in photon attenuation, loss of scattered photons, and increase in range of the secondary electrons. The magnitude of these perturbations depends on the extent and density of the inhomogeneity, the beam energy, field size, and depth.^5^,^8^–^11^ Various authors have investigated the accuracy with which calculation models can predict measured dose in lung equivalent material.^10^–^16^ As physically realistic analytical approaches become more practical for dose calculation, e.g., Monte Carlo,^17^,^18^ and convolution/superposition,^11^,^19^–^21^ there is a need for reliable dose assessment^22^ to validate them in various media densities.

A variety of measuring techniques used to investigate dose distribution are found in the literature. Common measuring devices include ionization chambers (both plane parallel and cylindrical), diodes, TLDs, diamond, and film. The use of film for measurements in solid water^23^–^26^ and low‐density inhomogeneities has been documented in several studies.^9^,^27^–^31^ Film is energy dependent and generally over‐responds at larger depths and field sizes though the magnitude of these effects varies across the literature.^32^–^35^ This dependence is usually attributed to the photoelectric process in the high atomic number component of the film layer, which rapidly becomes important for scattered low energy photons. Despite some limitations, film offers a convenient medium for easily generating profiles and two‐dimensional distributions. In addition to the popular Kodak XV film and the variety of other existing verification films,^36^–^39^ Kodak has recently released a new type of film that allows exposures at an extended dose range (EDR). The objective of this research is to provide a dosimetric evaluation of the new Kodak EDR film in homogeneous and lung density heterogeneous media.

The IC‐10 ionization chamber was chosen for an intercomparison with EDR film. Because of the relatively thin wall of this ion chamber, the inherent dosimetric difficulties arising from detector/media mismatch can be reduced even in electronic nonequilibrium conditions.^40^,^41^ This paper describes an investigation of the dependence of measured optical density on incident beam energy, field size, and depth. Investigations were conducted for two photon energies, 6 and 15 MV and covered a clinically relevant range of field sizes and depths in hetereogeneous slab phantoms.

## MATERIALS AND METHODS

The EDR film profile and depth dose responses were compared to ionization measurements due to the uniform energy response of ionization detectors in both homogeneous water and heterogeneous water‐lung phantoms.^40^ The sensitometric response of EDR film was also studied. All experiments were performed with 6 and 15 MV photon beams produced from a Varian Clinac 21‐EX (Varian Associates, Palo Alto, CA). Field sizes defined at 100 cm from the source for these measurements ranged from $2\times 2{\;\text{cm}}^{2}$ up to $25\times 25{\;\text{cm}}^{2}$. All measurements were carried out with a fixed source‐surface distance (SSD) of 90 cm for both photon energies.

### A. Phantom

**Figure 1:**
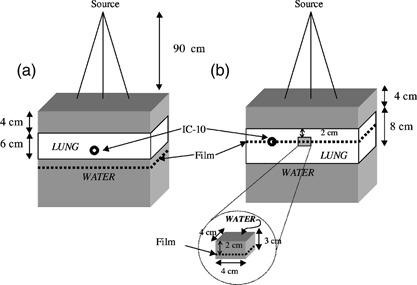
A schematic view of the experimental setup of the layer‐lung [(a), full‐slab and (b), tumor] geometry used in the measurement of dose.

The three phantoms used for this investigation included homogeneous solid water slabs of density 1.015 g/cm^3^ (Gammex RMI, Middleton WI) and two heterogeneous water‐lung equivalent phantoms (full slab and lung tumor geometry) pictured in Fig. 1. The total phantom size was at least 30 cm square (ranging up to 40 cm×40 cm for some slabs) by 30 cm thick. For the full slab heterogeneous phantom [(Fig. 1a)], the phantom material from depths of 4 to 10 cm was replaced with a 6 cm thick lung‐equivalent full slab phantom of density 0.300 g/cm^3^ (Gammex RMI, Middleton WI). The lung tumor heterogeneous phantom contained an 8 cm slab of lung material at a depth of 4 cm in solid water with a 4 cm square by 3 cm thick water equivalent block, mimicking a tumor, embedded inside a lung at 2 cm away from the proximal water/lung interface [(Fig. 1b)]. For this lung tumor phantom, smaller pieces of water and lung material, available in various sizes, were arranged to form the phantom. The solid water “tumor” was centered on the central axis of the beam. In this configuration, interfaces between the solid water and the lung material occurred both perpendicular and parallel to the beam axis [(Fig. 1b)]. Experiments on an analogous tumor geometry have been conducted by Rice *et al.* ^22^ Recesses were machined in both lung and water phantoms for the IC‐10 chamber. The Scanditronix‐Wellhöfer water phantom system (Scanditronix‐Wellhöfer, Uppsala, Sweden) was also used for the ionization measurements of profiles in homogeneous water.

### B. Film exposure and analysis

Double emulsion layered Kodak Ready‐Pack EDR2 film (Eastman Kodak Co., Rochester, NY) was used throughout the study. Film boxes were issued from the same batch. The films were sandwiched between either solid water and/or lung‐equivalent material slabs. Each film pack was pinpricked to avoid unwanted air in the envelope. Films were processed with a Kodak X_Omat‐3000 RA, automatic film processor with a 90 sec processing time. A few test films were developed prior to the start of the processing in order to stabilize the processing conditions. The developer temperature was stable at 99°F throughout the process. Unexposed films were processed at regular intervals and had their optical densities checked to monitor the stability of the processor. The film data were digitized using the Lumiscan 75 laser film scanner (Lumisys, Sunnyvale, CA) with a 0.025 cm/pixel, 12 bit resolution.

A series of sensitometric curves were generated in solid water for 6 and 15 MV photons at depths of maximum dose $\left(d_{\max}\right)$, 5, and 15 cm for field sizes of 3×3, 10×10, and $25\times 25{\;\text{cm}}^{2}$. Monitor units (MU) ranged from 0 to 500 MU in 12 to 14 increments. All films were irradiated in the “perpendicular” geometry with the radiation beam incident at right angles to the surface of the solid water phantom. For films irradiated for sensitometric analysis, optical densities were read out using a Digital Densitometer II (Sun Nuclear Corporation, Melbourne, FL) optical densitometer. The uncertainty associated with the optical density reader with respect to linearity and stability was estimated to be within 1%. The variation in optical density within a set of eight films, each irradiated and processed identically, was found to be approximately 2% (1σ). Net optical densities (OD) were read and plotted against dose to generate sensitometric curves; the net OD was defined as the OD read by the densitometer corrected for the background base plus fog (0 MU film). Dose for a given energy, field size, and depth was determined from the dosimetric beam parameters and the calibration factor relating the output of the linac. The calibration factor (0.8 cGy/MU) is determined at calibration conditions:^42^ 90 cm SSD, $10\times 10{\;\text{cm}}^{2}$ at a depth of 10 cm in water. Net optical densities for a dose of 50 cGy to the film were extracted from the sensitometric data for the different field sizes, depths, and energies for comparison purposes; the data reported are normalized to those from the $10\times 10{\;\text{cm}}^{2}$ field at a depth of 5 cm for the given energy.

Films irradiated for depth dose and profile analysis were also scanned and transformed to digitized images using the Lumiscan 75 laser film scanner. These images were subsequently analyzed with the Fuji film analysis software, ScienceLab 98–Image Gauge (Fuji Photo film Co, Ltd. Itasca, IL). The conversion from optical density to dose for all depth dose and profile measurements was accomplished using sensitometric curves at a depth of 5 cm for the $10\times 10{\;\text{cm}}^{2}$ field of each energy. In this conversion scenario no attempt was made to correct for the depth and field size dependence of the optical density.

Central axis depth dose measurements in homogeneous solid water and for the full‐slab lung‐water geometry were conducted for field sizes of 2×2, 3×3, 10×10, and $25\times 25{\;\text{cm}}^{2}$. Profiles at depths inside and beyond the lung slab (8 and 12 cm, respectively) were obtained in the full‐slab lung geometry for the aforementioned field sizes. Profile measurements at depths of $d_{\text{max}}$, 5 cm and 10 cm in homogeneous solid water were conducted for field sizes of 3×3, 10×10, and $25\times 25{\;\text{cm}}^{2}$. The central axis measurements in the heterogeneous full slab geometry were also displayed as lung dose correction factors. The correction factor is defined at a given depth on the central axis as the dose in the heterogeneous phantom divided by the dose in a solid water phantom.

Additional profiles at a depth of 8 cm were obtained in the lung tumor geometry for the 2×2, 5×5, 10×10, and $20\times 20{\;\text{cm}}^{2}$ field sizes. This measuring depth corresponds to a plane intersecting the tumor within the lung. All measurements were carried out with the film in the “perpendicular” geometry within the phantom. EDR films were irradiated with 175 MU each time to examine the dose in the extended range.

### C. Ionization measurements

Depth dose and profile measurements were conducted with an ionization chamber analogous to those performed with EDR film. The IC‐10 (Wellhofer Dosimetrie, Germany) ionization chamber with an outer and inner diameter of 6.8 and 6.0 mm, respectively (wall thickness of 0.4 mm and effective density of 1.76 g/cm^3^) was chosen for the dosimetric comparison. This corresponds to a wall of 70 mg/cm^2^ for the IC‐10, which is comparable to the Farmer‐type chamber (65 mg/cm^2^) used by Rice *et al.* ^40^ The charge was collected with a PRM Model SH‐1 (Precision Radiation Measurements, Tennessee) electrometer operated at 300 V. The IC‐10 was inserted into the phantom along the central axis of the beam at depths ranging from 1 to 20 cm in solid water in order to generate depth doses. The chamber was aligned with the field crosshair lines. The effective point of measurement^42^ of the chamber was taken into consideration for the depth positioning (upstream by 1.8 mm). Profiles for the homogeneous situation were measured with the IC‐10 chamber within the water tank. Profiles for the full‐slab lung heterogeneous phantom were obtained from the IC‐10 chamber positioned at off‐axis intervals of 0.5 cm and smaller steps of 0.2 cm in the fall‐off region by translating the chamber perpendicular to the electrode axis and using millimetric paper for the alignment with the field crosshair lines. Such displacement precision has been estimated to be within 1 mm (2σ). Profiles were obtained in the lung tumor phantom by rearranging the phantom pieces to fill every layer and prevent any loss of scatter. The uncertainties for the IC‐10 chamber, based on the reproducibility of readings repeated up to three times, were less than 1%. The measurement sessions lasted a few hours. Some of the readings taken at the beginning of the session were repeated at the end to estimate any possible drift in output of the linac or chamber sensitivity. These differences were less than 1%.

### D. Normalization

Depth dose curves in homogeneous solid water for both EDR film and IC‐10 were normalized to a common value at a depth of 5 cm for a given field size. The choice of normalization point will directly affect the interpretation of the dose distribution for the comparison. The homogeneous depth dose curves scaled in such manner were further used for normalizing the depth dose curves for the heterogeneous full‐slab lung situation. The uncertainties in the lung slab region due to electronic disequilibrium prevented the selection of a suitable normalization point near this area. In the full‐slab lung‐water case, the depth dose curves for a given detector were normalized to the value at a depth of 2 cm of the homogeneous phantom at fixed field sizes and energies. Profiles for both homogeneous and full‐slab lung‐water phantoms were normalized to their respective central axes. Profiles for the lung tumor geometry for a given energy and detector were normalized to the value at depth of 2 cm for a $2\times 2{\;\text{cm}}^{2}$ field on the homogeneous phantom. The low‐energy scatter contribution is relatively small for this depth and field combination.

## RESULTS AND DISCUSSION

### A. Film optical response

**Figure 2:**
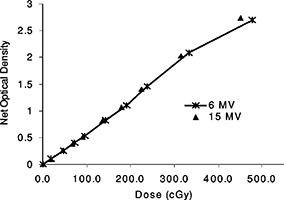
Sensitometric curves for EDR2 films for 6 and 15 MV photons, for a $10\times 10{\;\text{cm}}^{2}$ field size at 5 cm depth in the phantom.

**Figure 3:**
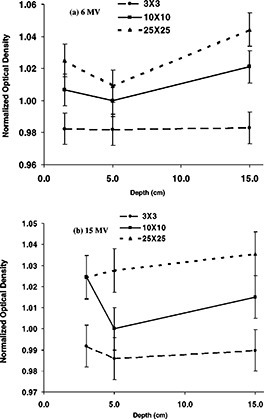
Normalized net optical densities for EDR film at a dose of 50 cGy as a function of field size and depth in solid for (a) 6 MV, and (b) 15 MV photons. The optical densities were normalized to the value for a $10\times 10{\;\text{cm}}^{2}$ field size at a depth of 5 cm. The uncertainties were estimated to 2% (1σ).

Figure 2 shows the sensitometric curves for the EDR film irradiated by 6 and 15 MV for a $10\times 10{\;\text{cm}}^{2}$ field size, at a depth of 5 cm in solid water. EDR film is found to have a linear response with dose, from 0 to 350 cGy for both energies. In this range the response of EDR film for the 15 MV beam is slightly higher than the 6 MV beam by 1–2%. In Fig. 3, the variation in optical density with depth as a function of field size is shown for a dose level of 50 cGy for both 6 MV [(Fig. 3a)] and 15 MV [(Fig. 3b)] beams. Field sizes of 3×3, 10×10, and $25\times 25{\;\text{cm}}^{2}$ are included in this figure. The plots are normalized to the net optical density for a $10\times 10{\;\text{cm}}^{2}$ field at depth of 5 cm. The variations of the optical densities with depth are on the order of 1% lower for the $3\times 3{\;\text{cm}}^{2}$ for both energies [(Figs. 3a)and (3b)]; these variations with depth are small for the $3\times 3{\;\text{cm}}^{2}$ field as compared to those observed with the $10\times 10{\;\text{cm}}^{2}$ and $25\times 25{\;\text{cm}}^{2}$. The variations with depth were in the order of 2–4% for the $25\times 25{\;\text{cm}}^{2}$ field for both energies; the highest difference 4.5% was reached at depth of 15 cm for the 6 MV beam for this field size. This behavior is a result of the softening of the beam at larger depths and field sizes which causes the over‐response of the film. The optical densities relative to the normalization point were 2% lower for the $3\times 3{\;\text{cm}}^{2}$ and 1–2% higher for the $25\times 25{\;\text{cm}}^{2}$ field. These differences can be explained if we consider that the $3\times 3{\;\text{cm}}^{2}$ field has a higher primary photon‐to‐scatter photon ratio and more beam hardening effect with depth than the 25×25 cm^2,23^ A similar trend was also reported with XV film.^25^,^35^ A detailed description of the sensitometric response of both EDR and XV films is presented in a recent publication.^43^

### B. Homogeneous phantom

**Figure 4:**
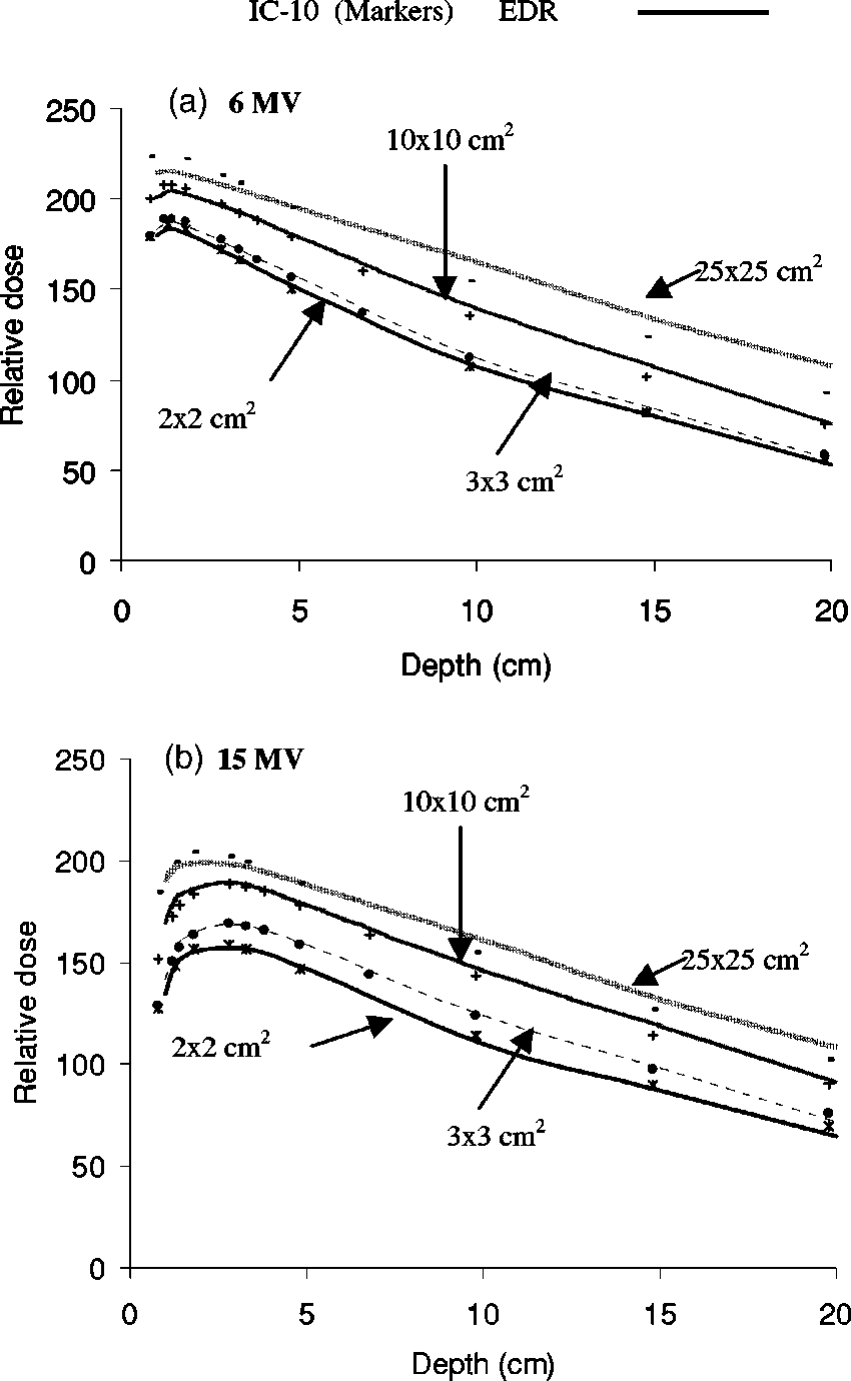
Depth dose comparison in homogeneous solid water between the EDR film in perpendicular orientation and the IC‐10 ionization chamber for different field sizes (2×2, 3×3, 10×10, and $25\times 25{\;\text{cm}}^{2}$) from (a) 6 MV, and (b) 15 MV photon beams. Depth dose curves for both dosimeters are normalized to a common value at a depth of 10 cm for each field size.

Figure 4 illustrates depth doses for 2×2, 3×3, 10×10, and $25\times 25{\;\text{cm}}^{2}$ field sizes for both 6 and 15 MV. Depth doses measured with EDR film showed agreement within 2% with IC‐10 measurements for all field sizes except for the $25\times 25{\;\text{cm}}^{2}$. For this large field, differences on the order of 4.2% and 1.6% were observed around the maximum dose for 6 MV [(Fig. 4a)] and 15 MV [(Fig. 4b)], respectively. The results indicate an under‐response of the EDR film data relative to IC‐10 data in this case. The choice of joint normalization of these two detectors at a depth of 5 cm implies discrepancies near the buildup area and a divergence further away in depth. Differences at large depths for the $25\times 25{\;\text{cm}}^{2}$ reached 8.4% and 3.9% at a depth of 15 cm for 6 MV [(Fig. 4a)] and 15 MV [(Fig. 4b)], respectively. This over‐response of EDR film with depth for the largest field is consistent with the observation in Fig. 3 of the rise in optical density response for a larger field and depth. The amount of over‐response does not map exactly to the same value due to differences in the analysis procedure but it is close within uncertainties. The forced joined normalization at depth of 5 cm and comparing to ionization measurements as opposed to film itself contribute to the discrepancy. Furthermore, the over‐response of the film at depth is more important for the lowest energy (6 MV), which has a higher scatter‐to‐primary ratio than the 15 MV photons. This is in agreement with the sensitometric response observed in Fig. 3. The agreement obtained between the EDR film in “perpendicular” geometry and the IC‐10 chamber are in agreement with the results obtained by Chetty and Charland^43^ in a “parallel” orientation of the film, except for the $25\times 25{\;\text{cm}}^{2}$ field. In the parallel orientation, these authors^43^ observed the following differences for the $25\times 25{\;\text{cm}}^{2}$ field in the buildup region and at 17 cm depth respectively: 1.8% and 2.9% for a 6 MV beam, and 0.9% and 4.6% for a 15 MV beam. No firm conclusion can be derived to explain the discrepancies between the results in parallel and perpendicular orientation. The experimental errors based on intrasession reproducibility were estimated to be 2%. It can be hypothesized that additional experimental uncertainties are to be expected from using the film in the parallel orientation; uncertainties are increased due to beam attenuation in the film and air gaps on either side of the film. In this case, the parallel orientation of the film appears to more closely agree with the ion chamber measurements.

**Figure 5:**
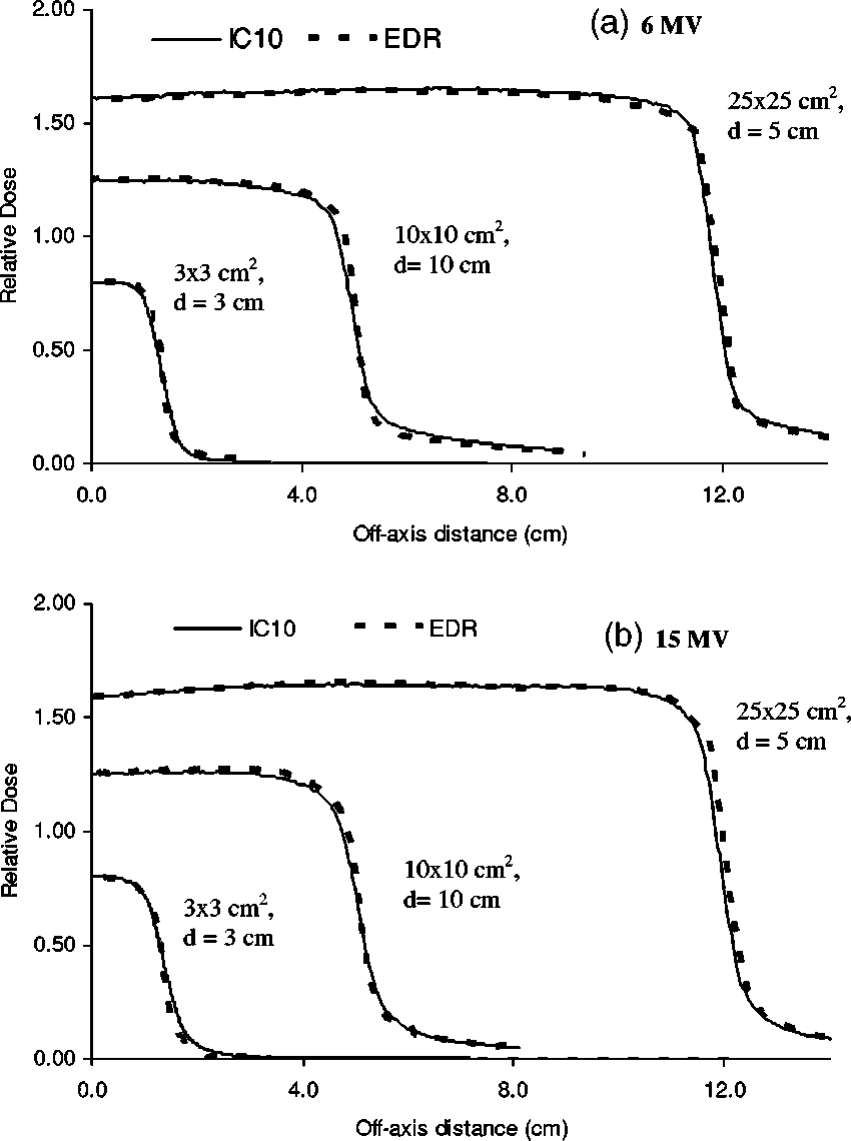
Profile comparison between the EDR film in homogeneous solid water and the IC‐10 ionization chamber in a water tank for different field sizes (2×2, 3×3, 10×10, and $25\times 25{\;\text{cm}}^{2}$) from (a) 6 MV, and (b) 15 MV photon beams. Profiles are normalized to their respective central axes. The IC‐10 profiles are subsequently rescaled to the central axis value of the EDR profiles for a given field size and energy.

Figure 5 illustrates the 6 and 15 MV beam profiles obtained in solid water for 3×3, 10×10, and $25\times 25{\;\text{cm}}^{2}$ field sizes only at depths of $d_{\text{max}}$, 10 and 5 cm, respectively. The discussion for this subset of data applies to the remaining profiles measured in this study. Similar results were the subject of a previous publication.^43^ Profiles obtained with film were clearly steeper than those measured with the ionization chamber (Fig. 5). This was most visible with the smallest field sizes $\left(3\times 3{\;\text{cm}}^{2}\right)$. These results are consistent with the description made by Chang *et al.* ^44^ Their computer simulation showed that the broadening of penumbra is almost zero when the detector is much smaller than the inherent penumbra, gradually increases when the detector size is comparable in size to the inherent penumbra and finally rises linearly with the detector size when the detector is much larger than the inherent penumbra. The 80–20% penumbra has been calculated from the profiles. The profile distributions were first normalized to 100% at the center using the slope of the penumbra extracted around the field edges. In our results, the penumbras for the 3×3, 10×10, and $25\times 25{\;\text{cm}}^{2}$ field sizes are estimated to be 3.2, 4.5, and 6.2 mm for the 6 MV beam and to 3.7, 6.0, and 7.0 mm for the 15 MV beam, respectively. These penumbral widths for the $3\times 3{\;\text{cm}}^{2}$ are small compared to the 6 mm diameter sensitive volume of the IC‐10 chamber. No off‐axis spectrum softening influence could otherwise be determined for the EDR film.

### C. Heterogeneous full‐slab lung phantom

**Figure 6:**
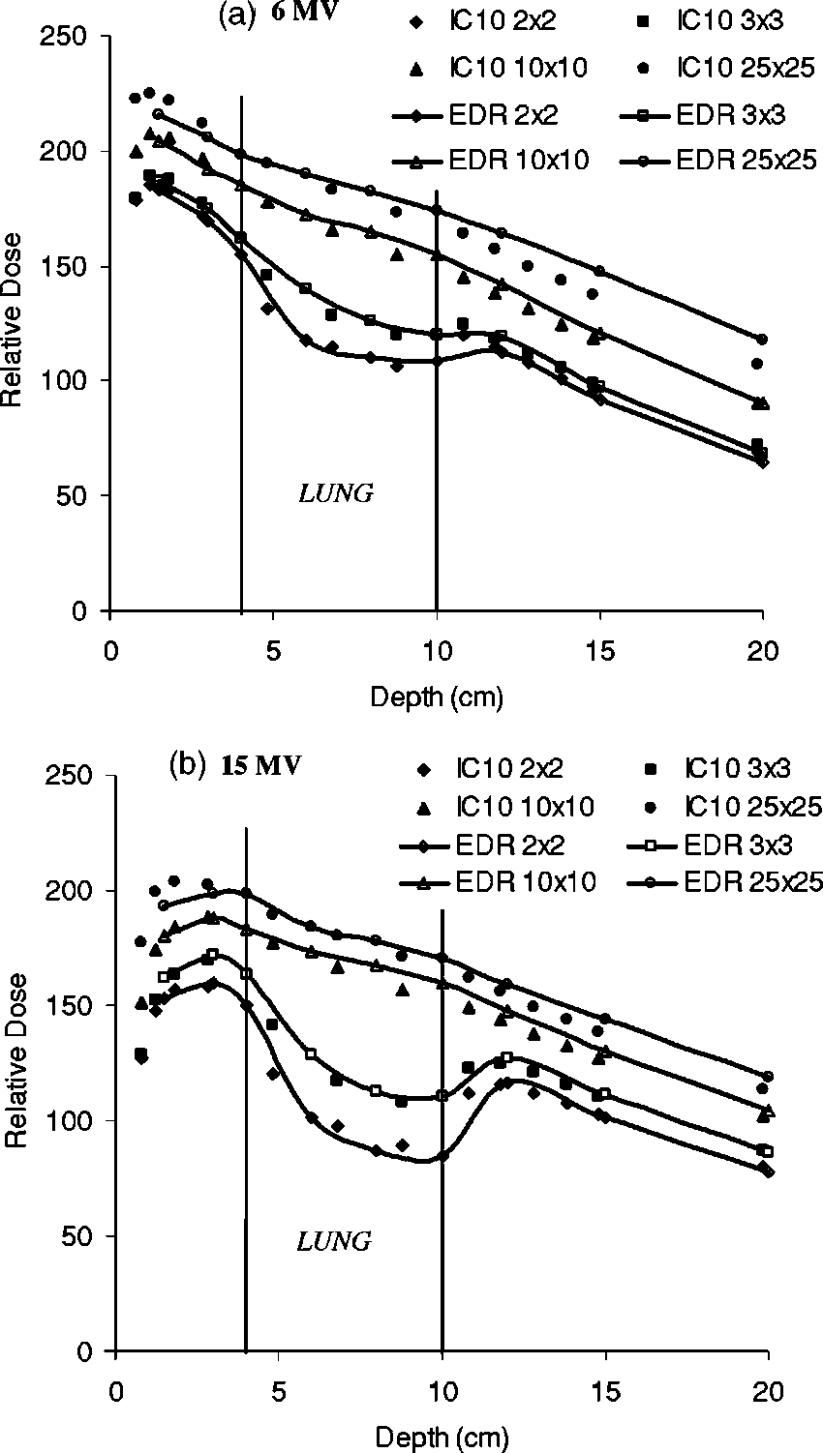
Depth dose comparison between the EDR film in the perpendicular orientation and the IC‐10 ionization chamber for different field sizes (2×2, 3×3, 10×10, and $25\times 25{\;\text{cm}}^{2}$) from (a) 6 MV, and (b) 15 MV photon beams in heterogeneous solid water and full‐slab lung. Depth dose curves for a given dosimeter are normalized to 2 cm depth of their analogous homogeneous depth doses for the respective field sizes.

Depth doses in the full‐slab lung‐water layered phantom are shown in Fig. 6 for 2×2, 3×3, 10×10, and $25\times 25{\;\text{cm}}^{2}$ field sizes and both 6 and 15 MV beams. The dose reduction inside the lung between depths of 4 and 10 cm is more apparent for the smaller fields. The dose is also slightly overestimated by the EDR film as compared to the IC‐10 for the most part; the differences are up to 2%. A possible explanation for the lower response of the IC‐10 in lung when lateral electronic equilibrium does not exist (for small field sizes) is its large integrating volume. A dose gradient within the chamber would result in an averaged reading. This, however, could only partly explain the difference between the film and the chamber response since the discrepancy existed well within lung, where the dose gradient was expected to be smaller. All depth doses in the heterogeneous lung slab geometry (Fig. 6) are higher than those in the homogeneous situations (Fig. 4) at depths beyond the build up on the distal side of the lung slabs. This is caused by the reduced radiological depth in lung which increases the primary fluence.10 The disagreement between EDR film and IC‐10 depth dose data for the $25\times 25{\;\text{cm}}^{2}$ increases in the buildup region, as it did for the homogeneous case, and further inside the lung. The normalization of these data was performed at a depth of 2 cm using the value from the homogeneous normalized depth dose. The over‐responses at depth of the larger field observed in the depth dose of the homogeneous phantom (preceding section) are again evident in Fig. 6 (7.4% and 3.7% at 15 cm depth for 6 and 15 MV respectively). These limitations of the film dosimeter were expected due to the increased film response at the lower end of the spectrum. No correction was applied to dose for sensitivity changes in the film associated with spectral changes.

**Figure 7:**
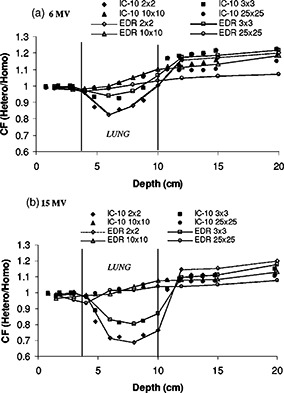
Lung correction factors vs depth for 2×2, 3×3, 10×10, and $25\times 25{\;\text{cm}}^{2}$ field sizes. A comparison between the EDR film in the perpendicular orientation and the IC‐10 ionization chamber (a) 6 MV, and (b) 15 MV photon beams in heterogeneous solid water and full‐slab lung. The correction factor at a given depth, field size, and energy is defined as the ratio of the dose in the heterogeneous phantom to the dose in the homogeneous phantom.

In Fig. 7, the lung correction factors (CF) are displayed for 2×2, 3×3, 10×10, and $25\times 25{\;\text{cm}}^{2}$ field sizes and both 6 and 15 MV beams. The lung correction factor as measured by a given detector is defined as the ratio of the dose in the heterogeneous lung phantom to the dose in the homogeneous water phantom at the same physical depth and for the same irradiation conditions. The lung correction factors can be seen as an attempt to correct the heterogeneous lung phantom data for the spectral behavior observed in the homogeneous case. The decrease in correction factor is more pronounced for the small field and the highest energy [(Fig. 7b)]. For the 15 MV beam and the smallest field, the dose reduction is associated mainly with the lack of electronic equilibrium. Published experiments in different configurations have shown these effects.^11^,^22^ The correction factor generally increases with field size inside the lung and decreases beyond it for both beams. The larger ratio of scatter‐to‐primary of the 6 MV beam as compared to 15 MV results in slightly different CF behavior. The dose reduction in lung for the 6 MV beam is largely due to reduction of scatter in the low density material. Similar results were observed for 4 and 15 MV by Rice *et al.* ^22^ When comparing the IC‐10 to the EDR film, we observed that the film gave a higher response inside the lung for $3\times 3{\;\text{cm}}^{2}$ 6 MV field. This over‐response gradually decreased with increasing field size. The case of the $2\times 2{\;\text{cm}}^{2}$ field was ambiguous and the agreement can be said to be within experimental error. This pattern of over‐response with field size was less severe for 15 MV; the agreement between IC‐10 and EDR is generally good inside the lung. The situation is reversed for the 6 MV beam at a depth beyond the lung in solid water where the response of the EDR film is now lower than the IC‐10. The under‐response of the EDR film in that region, increases with field size. At 15 MV, there is no such remarkable under‐response of the film in solid water beyond the lung.

While many authors have used film in combination with either TLDs or ionization measurements in lung‐water heterogeneous rectilinear^13^,^29^,^30^ and anthropomorphic phantoms,^15^,^16^ comprehensive comparison studies with film are limited. In a similar experiment at much higher energy (50 MV), Blomquist and Karlsson31 noticed an over‐response (4–6%) of the ion chamber within cork. These authors were, however, using an ionization chamber with a thick graphite wall of $160{\;\text{mg cm}}^{-2}$. Hence, increased photon attenuation of the wall compared to cork was expected. They also observed an under‐response of the ion chamber relative to the film in solid water beyond the cork. The under‐response of the chamber beyond the cork was more prominent for smaller fields. It was hypothesized that the deviations could be due to a combination of the volume averaging of the chamber and spectral changes at an interface. At such high energy it is the increased amount of pair production that could have some effect on the spectral dependence of the film. In our situation, the core of the discrepencies between the film and the chamber which occurs for the 6 MV field are believed to be due to spectral effects, as opposed to chamber wall effects. The low‐energy scatter dependence previously seen in the homogeneous situation is accentuated in the correction factor. More of the primary beam is being transmitted through the lung slab, as observed previously in the depth doses (Fig. 6) and hence less over‐response of the film. By taking the ratio, heterogeneous situation to the homogeneous situation, we observe a lower response of the film beyond the slab. The behavior of the correction factor inside the lung can be explained by a similar argument except that in this case there was less contrast in response, relative to the ion chamber, between the homogeneous and heterogeneous situations. The unexplained inverted response for the correction factor of the EDR inside lung for the $2\times 2{\;\text{cm}}^{2}$ field as well as some small variations are within experimental uncertainties.

**Figure 8:**
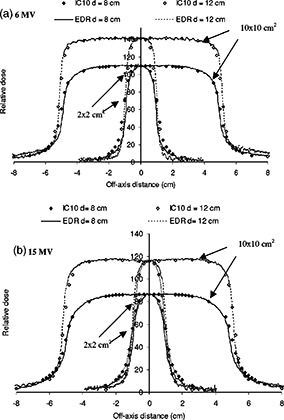
Profile comparison between the EDR film and the IC‐10 ionization chamber in heterogeneous full‐slab lung geometry for $2\times 2{\;\text{cm}}^{2}$ and $10\times 10{\;\text{cm}}^{2}$ fields at depth of 8 cm (in the lung) and 12 cm (beyond the lung). Profiles are shown for (a) 6 MV and (b) 15 MV photon beams. Profiles are normalized to their respective central axes. The IC‐10 profiles are subsequently rescaled to the central axis value of the EDR profiles for a given field size and energy.

Figure 8 shows a comparison between EDR film and IC‐10 profile data for 2×2 and $10\times 10{\;\text{cm}}^{2}$ field sizes and depths of 8 cm (inside lung) and 12 cm (distal side of lung inhomogeneity) for 6 and 15 MV beams. The lateral spreading of the penumbra inside lung can be seen in this figure; the profiles are broader upstream inside the lung (d=8 cm) than deeper in solid water at d=12cm. The penumbral width is affected by the electron energy and photon scatter within the medium: it is broadened within the lung and improves beyond it upon reentering solid water. The agreement between EDR film and IC‐10 measurements in the profile penumbral region is slightly better inside the lung than on the distal side of it. This is due to the detector size effect^44^ of the IC‐10, also seen previously in homogeneous water, being less pronounced inside the lung where the inherent penumbral width is increased as compared to downstream from the lung slab. It is also more prominent for the smallest field size ($2\times 2{\;\text{cm}}^{2}$). In (Fig. 8a), the penumbral width for a $2\times 2{\;\text{cm}}^{2}$ field as measured with EDR film is estimated to be 5 mm inside the lung (d=8 cm) and only 3.0 mm beyond the lung (d=12 cm). The corresponding penumbra values for the 15 MV beam [(Fig. 8b)] are 6.5 mm inside the lung (d=8 cm) and reduced to 4.2 mm beyond the lung slab (d=12 cm).

### D. Heterogeneous lung tumor phantom

**Figure 9:**
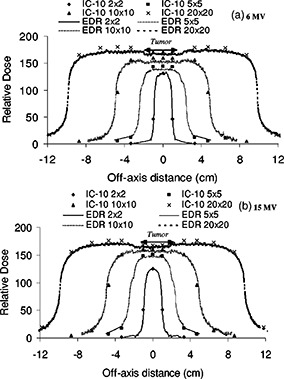
Profile comparison between the EDR film and the IC‐10 ionization chamber in the heterogeneous lung tumor geometry for 2×2, 5×5, 10* 10, and $20\times 20{\;\text{cm}}^{2}$ fields. The depth for measurement is 8 cm in the phantom; at this depth lung/tumor/lung interfaces are encountered laterally. Two photon beams are illustrated: 6 and 15 MV. Profiles are normalized to the 2 cm depth dose value for a $2\times 2{\;\text{cm}}^{2}$ field of the homogeneous phantom depth dose for fixed energy and field size.

A lung tumor geometry was tested for an examination of the transition between dose in water and dose in lung along the lateral direction. Experiments with similar geometries have presented central axis measurements only.^22^,^30^ The profiles for the lung tumor geometry are shown in Fig. 9. The measuring depth at 8 cm is such that lung/water/lung interfaces are encountered laterally. Profile data for 2×2, 5×5, 10×10, and $20\times 20{\;\text{cm}}^{2}$ fields and both energies are illustrated. The 4 cm wide tumor is centered in the beam and an increase in attenuation is visible in the center of the larger field. The dose in the vicinity of the tumor, as compared to adjacent lung material, results from a combination of transport effects in addition to the inherent beam profile shape. These include: the increased attenuation of the primary beam in water, the reduced photon scatter from the lung, and the increased range of the electrons in the lung. The profiles for a given detector and energy were normalized to the 2 cm depth of the respective $2\times 2{\;\text{cm}}^{2}$ homogeneous depth dose curves. At such normalization depths and field sizes, the low‐energy scatter contribution is reduced, and with the corresponding reduction in film over response to the lower energy component of the dose deposited. The IC‐10 and the EDR film are in good accordance for the smaller fields; small deviations from the agreement are seen with the increase in field size. The maximum discrepancy observed was for the 6 MV beam, where a 4% over‐response of EDR film is seen on the central axis where the solid water mini‐phantom (tumor) is located. The discrepancy between EDR and IC‐10 with increased field size is consistent with the previous observations of film spectral dependence at depths for larger fields. If the profiles were scaled vertically, they would overlap within 1%.

## CONCLUSIONS

Film dosimetry is in general a convenient method to generate one‐ and two‐dimensional dose distributions. An additional advantage of film is the possibility to position it at a water‐lung slab interface. The EDR film is found to be sensitive to the low end of the photon spectrum. The net optical density for the 6 MV beam was 5% higher at a depth of 15 cm for a $25\times 25{\;\text{cm}}^{2}$ field when compared to the $10\times 10{\;\text{cm}}^{2}$ field and depth of 5 cm reference value. The consistency of the Kodak EDR film with the ionization measurements for dosimetry has been established in homogeneous and lung‐water heterogeneous phantoms for field sizes up to $10\times 10{\;\text{cm}}^{2}$. Differences between the IC‐10 and the EDR film at large depths in water for the $25\times 25{\;\text{cm}}^{2}$ reached 8% and 4% at depth of 15 cm for 6 and 15 MV respectively when normalization was at a depth of 5 cm. The corresponding over‐response at 15 cm depth for the $25\times 25{\;\text{cm}}^{2}$ in the heterogeneous lung phantom was lower than in the homogeneous case by less than a percent due to the relative increased primary transmission through the lung slab. The results of this study indicate that EDR

film is a flexible tool for relative dosimetry in higher dose ranges in both homogeneous solid water and water‐lung equivalent heterogeneous phantom for moderate field sizes and depths.

## ACKNOWLEDGMENTS

P. C. wishes to acknowledge Lori Paniak for helpul comments. We thank Bryan Bednarz for help with the revisions. This work has been supported in part by NIH Grant No. P01‐CA59827.

## References

[1] B. A. Fraass , K. Doppke , M. A. Hunt , G. J. Kutcher , G. Starkschall , R. Stern , and J. Van Dyk , “American Association of Physicists in Medicine Radiation Therapy Committee Task Group 53: Quality assurance for clinical radiotherapy treatment planning,” Med. Phys. 25, 1773–1829 (1998). 9800687 10.1118/1.598373

[2] J. M. Robertson , R. K. Ten Haken , M. B. Hazuka , A. T. Turrissi , M. K. Martel , A. T. Pu , J. F. Littles , F. J. Martinez , I. R. Francis , L. E. Quint , and A. S. Lichter , “Dose escalation for non‐small cell lung cancer using conformal radiation therapy,” Int. J. Radiat. Oncol., Biol., Phys. 37, 1079–85 (1997). 9169816 10.1016/s0360-3016(96)00593-7

[3] R. K. Ten Haken , M. K. Martel , M. L. Kessler , M. B. Hazuka , T. S. Lawrence , J. M. Robertson , A. T. Turrissi , and A. S. Lichter , “Use of Veff and iso_NCTP in the implementation of dose escalation protocols,” Int. J. Radiat. Oncol., Biol., Phys. 27, 689–95 (1993). 8226166 10.1016/0360-3016(93)90398-f

[4] T. R. Mackie , J. E. El‐Khatib , J. Battista , J. Scrimger , J. van Dyk , and J. R. Cunningham , “Lung dose corrections for 6 and 15 MV X‐rays,” Med. Phys. 12, 327–332 (1985). 3925308 10.1118/1.595691

[5] M. E. J. Young and R. O. Komelson , “Dose corrections for low‐density tissue inhomogeneities and air channels for 10‐MV x rays,” Med. Phys. 10, 450–5 (1983). 6888356 10.1118/1.595392

[6] A. K. Rustgi , A. Samuels , and S. N. Rustgi , “Influence of air inhomogeneities in radiosurgical beams,” Med. Dosim. 22, 95–100 (1997). 9243461 10.1016/s0958-3947(97)00001-0

[7] E. E. Klein , A. Morrison , J. A. Purdy , M. V. Graham , and J. Matthews , “A volumetric study of measurements and calculations of lung density corrections for 6 MV and 18 MV photons,” Int. J. Radiat. Oncol., Biol., Phys. 37, 1163–1170 (1997). 9169827 10.1016/s0360-3016(97)00110-7

[8] D. J. Dawson , J. M. Harper , and A. C. Akindradewo , “Analysis of physical parameters associated with the measurement of high‐energy x‐ray penumbra,” Med. Phys. 11, 491–7 (1984). 6434915 10.1118/1.595542

[9] R. O. Kornelson and M. E. J. Young , “Changes in the dose‐profile of a 10 MV x‐ray beam within and beyond low density material,” Med. Phys. 9, 114–16 (1982). 6804765 10.1118/1.595059

[10] E. El‐Khatib , M. Evans , M. Pla , and J. R. Cunningham , “Evaluation of lung dose correction methods for photon irradiations of thorax phantoms,” Int. J. Radiat. Oncol., Biol., Phys. 17, 871–878 (1989). 2777679 10.1016/0360-3016(89)90081-3

[11] T. R. Mack , J. W. Scrimger , and J. J. Battista , “A convolution method of calculating dose for 15 MV X‐rays,” Med. Phys. 12, 1888–196 (1985). 10.1118/1.595774 4000075

[12] K. E. Ekstrand and W. H. Barnes , “Pitfalls in the use of high energy x‐rays to treat tumors in the lung,” Int. J. Radiat. Oncol., Biol., Phys. 18, 249–252 (1990). 2105286 10.1016/0360-3016(90)90290-z

[13] M. A. Hunt , G. E. Desobry , B. Fowble , and L. R. Coia , “Effect of low‐density lateral interfaces on soft‐tissue doses,” Int. J. Radiat. Oncol., Biol., Phys. 37, 475–482 (1997). 9069324 10.1016/s0360-3016(96)00499-3

[14] M. R. Arnfield , C. H. Siantar , J. Siebers , P. Carmon , L. Cox , and R. Mohan , “The impact of electron transport on the accuracy of computed dose,” Med. Phys. 27, 1266–1274 (2000). 10902555 10.1118/1.599004

[15] M. J. Butson , R. Elferink , T. Cheung , P. K. N. Yu , M. Stokes , K. Y. Quach , and P. Metcalfe , “Verification of lung dose in an anthropomorphic phantom calculated by the collapsed cone convolution method,” Phys. Med. Biol. 45, 143–49 (2000). 10.1088/0031-9155/45/11/402 11098921

[16] W. U. Laub , A. Bakai , and F. Nüsslin , “Intensity modulated irradiation of a thorax phantom: comparisons between measurements, Monte Carlo calculations and pencil beam calculations,” Phys. Med. Biol. 46, 1695–1706 (2001). 11419628 10.1088/0031-9155/46/6/308

[17] M. Fippel , “Fast Monte Carlo dose calculation for photon beams based on the VMC electron algorithm,” Med. Phys. 26, 1466–1475 (1999). 10501045 10.1118/1.598676

[18] J. Sempau , S. J. Wilderman , and Alex F. Bielajew , “DPM, a fast, accurate Monte Carlo code optimized for photon and electron radiotherapy treatment planning dose calculations,” Phys. Med. Biol. 45, 2263–2291 (2000). 10958194 10.1088/0031-9155/45/8/315

[19] A. Ahnesjö , P. Andreo , and A. Brahme , “Calculation and application of point spread functions for treatment planning with high energy photon beams,” Acta Oncol. 26, 49–55 (1987). 3109459 10.3109/02841868709092978

[20] P W. Hoban , D. C. Murray , P. E. Metcalfe , and W. H. Round , “Superposition dose calculation in lung for 10 MV photons,” Australas. Phys. Eng. Sci. Med. 13, 81–92 (1990). 2375704

[21] M. Miften , M. Wiesmeyer , S. Monthofer , and K. Krippner , “Implementation of FFT convolution and multigrid superposition models in the FOCUS RTP system,” Phys. Med. Biol. 45, 817–833 (2000). 10795974 10.1088/0031-9155/45/4/301

[22] R. Rice , B. Mijnheer , and L. Chin , “Benchmark measurements for lung dose corrections,” Int. J. Radiat. Oncol., Biol., Phys. 15, 399–409 (1988). 3403321 10.1016/s0360-3016(98)90022-0

[23] J. L. Robat and B. G. Clark , “The use of radiographic film for linear accelerator stereotactic radiosurgical dosimetry,” Med. Phys. 26, 2144–2150 (1999). 10535631 10.1118/1.598730

[24] R. L. Stern , B. A. Fraass , A. Gerhardsson , D. L. McShan , and K. L. Lam , “Generation and use of measurement‐based 3‐D dose distributions for 3‐D dose calculation verification,” Med. Phys. 19, 165–173 (1992). 1620042 10.1118/1.596873

[25] L. J. van Battum and B. J. M. Heijmen , “Film dosimetry in water in a 23 MV therapeutic photon beam,” Radiother Oncol. 34, 152–159 (1995). 7597214 10.1016/0167-8140(94)01500-3

[26] N. A. M. van Bree , M. H. M. Izdes , H. Huizenga , and B. J. Mijnheer , “Film dosimetry for radiotherapy treatment planning verification of a 6 MV tangential breast irradiation,” Radiother. Oncol. 31, 251–255 (1994). 8066209 10.1016/0167-8140(94)90431-6

[27] P. Hoban , P. Keall , and W. Round , “The effect of density on the 10 MV photon beam penumbra,” Australas. Phys. Eng. Sci. Med. 15, 113–123 (1992). 1471961

[28] R. C. Miller , J. A. Bonner , and R. W. Kline , “Impact of beam energy and field margin on penumbra at tumor‐lung parenchyma interfaces,” Int. J. Radiat. Oncol., Biol., Phys. 41, 707–13 (1998). 9635723 10.1016/s0360-3016(98)00133-3

[29] R. Mayer , A. Williams , T. Frankel , Y Chong , S. Simons , N. Yang , and R. Timmerman , “Two‐dimensional film dosimetry application in heterogeneous materials exposed to megavoltage photon beams,” Med. Phys. 24, 455–460 (1997). 9089597 10.1118/1.598126

[30] E. Yorke , L. Harisiadis , B. Wessels , H. Aghdam , and R. Altemus , “Dosimetric considerations in radiation therapy of coin lesions of the lung,” Int. J. Radiat. Oncol., Biol., Phys. 34, 481–487 (1995). 10.1016/0360-3016(95)02036-5 8567352

[31] M. Blomquist and M. Karlsson , “Measured lung dose correction factors for 50 MV photons,” Phys. Med. Biol. 43, 3225–3234 (1998). 9832013 10.1088/0031-9155/43/11/005

[32] J. F. Williamson , F. M. Khan , and S. C. Sharma , “Film dosimetry of megavoltage photon beams: a practical method of intensity‐to‐isodose curve conversion,” Med. Phys. 8, 94–98 (1981). 7207433 10.1118/1.594913

[33] C Danciu , B. S. Proimos , J. Rosenwald , and B. J. Mijnheer , “Variation of sensitometric curves of radiographic films in high energy photon beams,” Med. Phys. 28, 966–974 (2001). 11439493 10.1118/1.1376443

[34] N. Suchowerska , P. Hoban , M. Butson , A. Davidson , and P. Metcalfe , “Directional dependence in film dosimetry: radiographic and radiochromic film,” Phys. Med. Biol. 46, 1391–1397 (2000). 10.1088/0031-9155/46/5/305 11384060

[35] J. R. Sykes , H. V. James , and P. C. Williams , “How much does film sensitivity increase at depth for larger fields?,” Med. Phys. 26, 329–330 (1999). 10076992 10.1118/1.598520

[36] C.‐W. Cheng and I. J. Das , “Dosimetry of high energy photon and electron beams with CEA films,” Med. Phys. 23, 1225–1232 (1996). 8839417 10.1118/1.597865

[37] P. Cadman , “Use of CEA TVS film for measuring high energy photon beam distributions,” Med. Phys. 25, 1435–37 (1998). 9725130 10.1118/1.598316

[38] W. L. McLaughlin , Y. Chen , C. G. Soares , A. Miller , G. V. Van Dyke , and D. F. Lewis , “Sensitometry of the response of a new radiochromic film dosimeter to gamma radiation and electron beams,” Nucl. Instrum. Methods Phys. Res. A 302, 165–167 (1995).

[39] P. J. Muench , A. S. Meigooni , R. Nath , and W. L. McLaughlin , “Photon energy dependence of the sensitivity of radiochromic film and comparison with silver halide film and LiF TLDs used for brachytherapy dosimetry,” Med. Phys. 18, 769–775 (1991). 1921886 10.1118/1.596630

[40] R. Rice , J. L. Hansen , L. M. Chin , B. J. Mijnheer , and B. E. Bjärngard , “The influence of ionization chamber and phantom design on the measurement of lung dose in photon beams,” Med. Phys. 15, 884–890 (1988). 3237146 10.1118/1.596171

[41] T. Mauceri and K. Kase , “Effects of ionization chamber construction on dose measurements,” Med. Phys. 14, 653–656 (1987). 3627005 10.1118/1.596034

[42] American Association of Physicists in Medicine, Radiation Therapy Committee Task Group 21 , “A protocol for determination of absorbed dose from high‐energy photon and electron beams,” Med. Phys. 10, 41–771 (1983).

[43] I. J. Chetty and P. Charland , “Investigation of Kodak Extended Dose Range (EDR) film for megavoltage photon beam dosimetry,” Phys. Med. Biol. 47, 3629 (2002). 12433124 10.1088/0031-9155/47/20/305

[44] K.‐S. Chang , F.‐F. Yin , and K.‐W. Nie , “The effect of detector size to the broadening of the penumbra–a computer simulated study,” Med. Phys. 23, 1407–1411 (1996). 8873038 10.1118/1.597724

